# Molecular Interaction Fields Describing Halogen Bond Formable Areas on Protein Surfaces

**DOI:** 10.1021/acs.jcim.4c00896

**Published:** 2024-07-16

**Authors:** Daichi Hayakawa, Yurie Watanabe, Hiroaki Gouda

**Affiliations:** Division of Biophysical Chemistry, Department of Pharmaceutical Sciences, Graduate School of Pharmacy, Showa University, 1-5-8 Hatanodai, Shinagawa-ku, Tokyo 142-8555, Japan

## Abstract

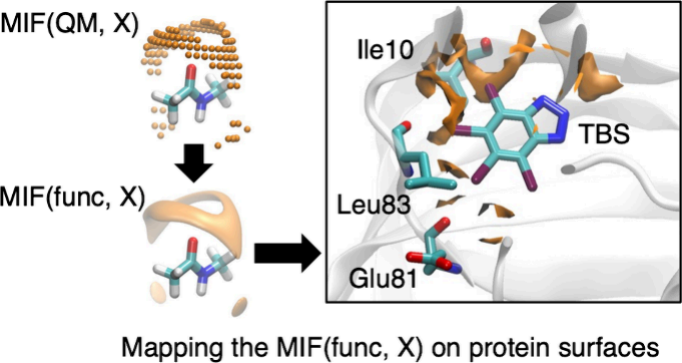

Molecular interaction fields (MIFs) are three-dimensional interaction maps that describe the intermolecular interactions expected to be formed around target molecules. In this paper, a method for the fast computation of MIFs using the approximation functions of quantum mechanics-level MIFs of small model molecules is proposed. MIF functions of *N*-methylacetamide with chlorobenzene, bromobenzene, and iodobenzene probes were precisely approximated and used to calculate the MIFs on protein surfaces. This method appropriately reproduced halogen-bond-formable areas around the ligand-binding sites of proteins, where halogen bond formation was suggested in a previous study.

## Introduction

Molecular interaction fields (MIFs) are three-dimensional (3D) interaction maps describing the intermolecular interactions expected to be formed around target molecules.^1,2^ MIFs can be investigated for various applications in the field of in silico drug design, including the exploration of ligand-binding sites in proteins,^3,4^ and construction of quantitative structure–activity relationship (QSAR) models.^5−7^ Generally, MIFs are calculated as intermolecular interaction energies between a target and probe molecule arranged at grid points defined around the target molecule. The energies at the grid points, that is the MIF energies, are used as descriptors or for constructing a contour map.^2^ Various molecules and functionalities can be used as probes, including water, methyl or hydroxyl groups, and amine nitrogen or carboxyl oxygen atoms.^1^ In the calculations, the intermolecular interaction energies are evaluated using potential functions based on molecular mechanics (MM).^3,4,8−15^ Furthermore, 3D interaction maps similar to MIFs can be calculated using empirical methods. SuperStar^16−19^ is an empirical method developed for calculating 3D interaction maps for small molecules and proteins using IsoStar,^20^ which is a knowledge-based library of intermolecular interactions constructed based on the Cambridge structural database^21^ and the Protein Data Bank (PDB) (www.rcsb.org).^22^ The 3D contour maps calculated by SuperStar are comparable to those obtained using MIF calculations.^16−20^ SuperStar predicts the statistical probabilities of interactions around the target molecules, whereas MIF calculations predict their interaction energies. Thus, MIF calculations and knowledge-based methods, such as SuperStar, are complementary.

The halogen bond, which is the second focal point of this study, is a noncovalent bond formed between a σ-hole on a halogen of a halogenated molecule and an electron-rich chemical group.^23−27^ Halogen bond strengths generally depend on the halogen atomic number (I > Br > Cl > F) and strongly depend on the orientations of the donor and acceptor.^24−27^ Additionally, fluorine atoms scarcely form halogen bonds because the σ–holes on fluorine atoms are generally small. Halogen bonds play an important role in molecular recognition in biological systems and are of significant interest in structure-based drug design (SBDD).^28,29^ Therefore, MIFs describing halogen bonds hold promise for in silico SBDD. However, conventional MM models are generally unsuitable for characterizing halogen bonds because they do not describe halogen interaction anisotropies. Currently, quantum mechanics (QM) calculations constitute the most reliable method for describing halogen bonds,^30−33^ although new-generation MM force fields are being actively studied.^34−41^ Therefore, QM calculations are desirable for evaluating MIFs, including those involving halogen bonds.

Zimmermann et al. proposed a QM-derived empirical scoring function for halogen bonds termed XBScore, which is similar to MIFs.^42^ Their approach entails the establishment of 3D halogen bond interaction maps of a model molecule (*N*-methylacetamide) termed the “spherical score,” and the orientation dependences of halogen bonds, referred to as the “sigma hole score”. The spherical and sigma-hole scores are combined to generate an XBScore.^42^ The spherical scores, being similar to MIFs, are obtained by calculating the interaction energies between the *N*-methylacetamide molecule and a halobenzene molecule arranged at spherical grid points around the former. The interaction energies at the grid points are independently determined by QM calculations. The obtained XBScores were validated for complexes of proteins and ligands bearing a halogenated benzene moiety registered in the PDB.

Inspired by the above-mentioned studies, we previously proposed a method for calculating MIFs that describe halogen bonds based on QM calculations.^43^ The QM-level MIF calculation method was successfully applied to small compounds and described halogen bonds, which makes it promising for drug design and QSAR studies that consider halogen bond effects. However, its application to large molecules, such as proteins, is unfeasible because of the limitations of current computational capabilities.

In this study, we propose a calculation method for determining the approximation functions of QM-level MIFs of small model molecules via Gaussian expansion. *N*-Methylacetamide was used as the model molecule to demonstrate the calculation. First, the QM-level MIFs were calculated for the *N*-methylacetamide model. To explore the halogen-bond-formable areas around *N*-methylacetamide, chlorobenzene, bromobenzene, and iodobenzene molecules were utilized as probes for the MIF calculations. The approximation functions were established based on the obtained QM-level MIFs. We demonstrate that the obtained approximation functions can precisely reproduce the QM-level MIFs of halogen bonds and significantly reduce the processing time. Furthermore, we propose a method for calculating approximated MIFs on protein surfaces using approximation functions. Using this approach, the MIFs on protein surfaces can be calculated within a practical computational time period. Furthermore, the proposed method was found to adequately describe halogen-bond-formable areas in the ligand-binding sites of proteins where halogen bond formation has been previously suggested. The validity of the calculation method was examined for 68 halogen bond sites in protein/ligand systems.

## Methods

### Halogen Bond Geometric Parameters

**Figure 1:**
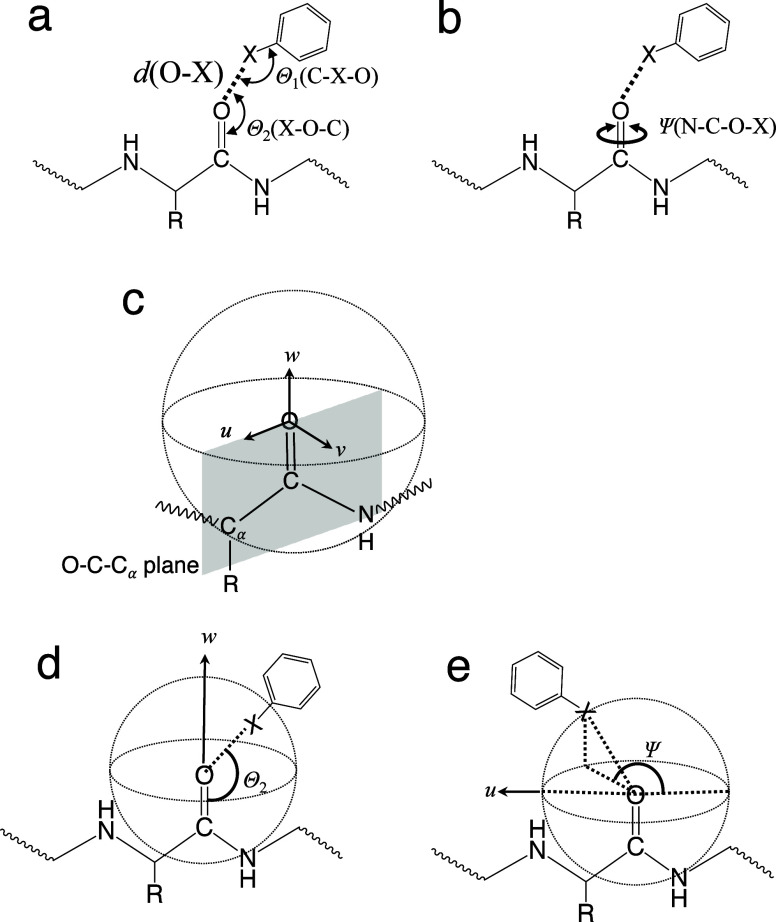
Definition of the geometric parameters of halogen bonds. (a) Definitions of *d*(O–X), Θ_1_(C–X–O), and Θ_2_(X–O–C). (b) Definition of Ψ(N–C–O–X). (c) The molecular coordinate system (*o-uvw*) in relation to an amino acid residue. The *w* axis is defined along the C=O axis of an amino acid residue. The u axis is defined on the O=C–C_α_ plane perpendicular to the *w* axis. The *v* axis is perpendicular to the *u* and *w* axes. (d) The Θ_2_(X–O–C) parameter in a spherical system; 180 – Θ_2_ corresponds to the polar angle (θ). (d) The Ψ(N–C–O–X) parameter in a spherical system; 180 + Ψ corresponds to the azimuthal angle (ϕ).

In protein interactions with halogenated molecules, halogen bonds predominantly form between the main chain carbonyl oxygens and the halogen atoms of the respective molecule.^28^ Therefore, the study was focused on the evaluation of this type of halogen bond. Based on a previous study, four geometrical parameters of such halogen bonds were defined, namely, the distance *d*(O–X), the angles Θ_1_(C–X–O) and Θ_2_(X–O–C), and the torsion angle Ψ(N–C–O–X), (Figure 1a,b).^28^ The molecular coordinates (*o-uvw*) for each amino acid residue were defined as shown in Figure 1c. The origin of the coordinate system is defined by the carbonyl oxygen with respect to each amino acid residue. The *w* axis is defined along the C–O bond. The *u* axis is defined on the O–C–C_α_ plane perpendicular to the *w* axis. The *v* axis is perpendicular to the *u* and *w* axes. When defining the molecular coordinates, the angles θ = 180 – Θ_2_ and ϕ = 180 + Ψ describe the polar and azimuthal angles, respectively (Figure 1d,e). The northern (90 < Θ_2_) and southern (90 > Θ_2_) hemisphere of amino acid residues are described using the angle Θ_2_. Additionally, directions parallel (Ψ ≈ 0, 180) and perpendicular (Ψ ≈ 90, 270) to the O–C–C_α_ plane are described. In this study, we adopted these parameters to describe the halogen bond geometries.

### MIF(QM, X) Calculations for the *N*-Methylacetatmide Model

**Figure 2:**
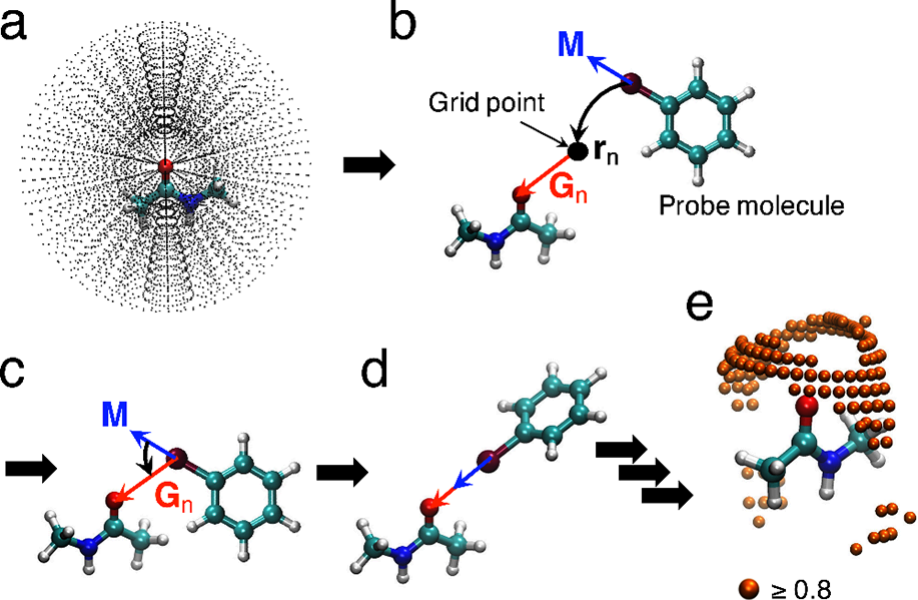
Schematic of the MIF calculation procedure. (a) Definition of grid points around a target molecule. An *N*-methylacetamide molecule is used as the target. (b) Positioning of probe molecule on a grid point. A vector (**G**_n_) is measured from the grid point to the carbonyl atom of the *N*-methylacetamide molecule. A molecular axis (**M)** is measured along the C-X bond of the probe molecule. (c) The probe molecule is rotated such that the molecular axis **M** coincides with vector **G**_n_. (d) The interaction energy between *N*-methylacetamide and a probe molecule is estimated by a QM calculation. (e) MIF(QM, X) is obtained by repeating the procedures described in (b) to (d). The normalized MIF(QM, X) energies at the grid points are represented by spheres of distinct colors depending on the values. Orange spheres (≥0.8) are shown as an example.

*N*-methylacetamide was deemed appropriate for modeling protein main chains because in protein/ligand systems, the carbonyl oxygens (O=C) of the protein main chains are primarily involved in halogen bond formation.^28^ The MIFs for *N*-methylacetamide with halogenated benzene probes (chlorobenzene, bromobenzene, and iodobenzene) were calculated based on our previously reported protocol (Figure 2).^43^ Spherical grid points were defined around the *N*-methylacetamide (Figure 2a). The molecular coordinates were defined in the same manner as those shown in Figure 1. In this approach, grid points were defined for the radial direction from 2 to 7 Å at 0.5 Å intervals, and for the polar and azimuth angles from 0° to 180° and from 0° to 360° at 10° intervals, respectively. The probe molecule was arranged on a grid point (Figure 2b). The vector **G**_n_ was defined from the grid point to the carbonyl oxygen atom of the *N*-methylacetamide; the vector **M** was defined on the halogen atom of the probe molecule along the C–X axis (Figure 2b). The probe molecule was rotated such that the M-axis was coincident with the vector **G**_n_ (Figure 2c). For the obtained geometry, the intermolecular interaction energies between *N*-methylacetamide and the probe molecule was calculated using QM calculations (Figure 2d).

The interaction energy was calculated using the ωB97X-D/aug-cc-pVDZ-PP level of theory for the bromobenzene and iodobenzene systems, or MP2/aug-cc-pVDZ for the chlorobenzene system.^44,45^ The basis set superposition error was corrected using the counterpoise method.^46^ Pseudopotentials were adopted for bromine and iodine atoms to consider relativistic effects, according to previous reports.^47−49^ The adopted QM calculations are validated in the following section. The interaction energies were calculated independently for each grid point (Figure 2e). The calculated energies were normalized to values ranging from 0 to 1 using the most stable energy. The normalized energies were defined as the MIF energies at the respective grid points. In addition, the MIF energies at the grid points with repulsive interaction energies were set to 0. In this study, the normalized MIF energy was regarded as the strength of the halogen bond at that point. The calculated MIFs are denoted as MIF(QM, X), where X represents Cl, Br, or I, indicating chlorobenzene, bromobenzene, or iodobenzene probe molecules, respectively.

### Determining Approximation Functions *E*_*X*_(r) for MIF(QM, X)

As mentioned, the above-discussed MIF calculations cannot be directly adopted for the ligand-binding sites of proteins due to computational limitations. Therefore, we attempted to derive the approximation functions, *E*_*X*_(**r**) (X = Cl, Br, I) for the MIFs(QM, X) of *N*-methylacetamide, and subsequently evaluate the approximated MIFs for proteins using *E*_*X*_(**r**). The approximation function *E*_*X*_(**r**) is expressed as a linear combination of Gaussian functions as follows:
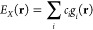
1where *c*_*i*_, *g*_*i*_ = *u*^*k*^*v*^*l*^*w*^*m*^exp(-α*r*^2^) (*k*, *l*, *m* = 0, 1, 2, ...), and **r** = (*u*, *v*, *w*) are the expansion coefficient, Cartesian Gaussian function (CGF), and position vector, respectively. The *c*_*i*_ coefficients are determined by minimizing the Δfunction (eq 2):
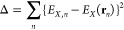
2where *n*, *E*_*X,n*_, and **r**_*n*_ are the indices of the grid point, MIF(QM, X) energy of grid point n, and position vector of the grid point, respectively. To minimize Δ, we set the first derivative equal to zero (eq 3).
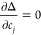
3

By adding eqs 1 and 2 to eq 3, the following relationship is obtained:
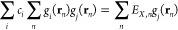
4Defining the relations,
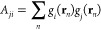
5a
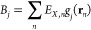
5b

eq 4 is represented by eq 6.
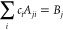
6eq 6 is represented by matrices **A**, **B**, and **c**, which consist of elements A_ji_, B_j_, and *c*_*i*_, respectively:
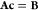
7

Using the above relation, we can obtain coefficients *c*_*i*_ by minimizing the Δfunction. The MIFs obtained by the approximation function *E*_*X*_(r) are denoted as MIF(func, X).

Using this method, the approximation function can be obtained in a single calculation. The proposed method differs from the previously described SuperStar method^17^ in that it uses high-order CGFs to obtain a highly accurate approximation. In the XBScore approach, discrete sampled data is applied directly to obtain spherical scores,^42^ whereas our method relies on sampled data to determine and subsequently apply approximation functions. The proposed method is highly accurate and presents versatile and expandable applicability.

### Mapping of MIF(func, X) on Protein Surfaces

**Figure 3:**
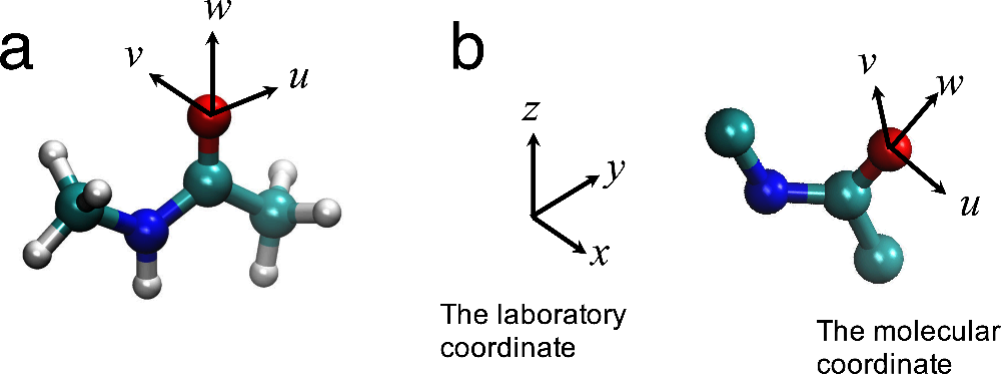
(a) *u*, *v*, and *w* axes of the molecular coordinate in relation to the *N*-methylacetamide model. The *w* axis is defined along to the C=O axis of *N*-methylacetamide. The *u* axis is defined on the O=C–C plane of *N*-methylacetamide, perpendicular to the *w* axis. The *v* axis is perpendicular to the *u* and *w* axes. (b) *x*, *y*, and *z* axes of the laboratory coordinate and *u*, *v*, and *w* axes of the molecular coordinate defined on an amino acid residue.

We aimed to evaluate the approximated MIFs(func, X) of proteins using the approximation function *E*_*X*_(r), which is determined as described above. The procedure for calculating the MIFs(func, X) of proteins from *E*_*X*_(r) is referred to as “mapping.” The molecular coordinates *o-uvw* define the grid points of the MIFs(QM, X) (Figure 3a), and consequently, the function *E*_*X*_(r). On the contrary, amino acid residues in proteins with random orientations are represented using laboratory coordinates (*o*-*xyz*). In the present study, the *x*, *y*, and *z* components of the position vectors of the atoms constituting proteins and ligands in the laboratory coordinate system correspond to the atomic coordinates listed in the PDB format files. The grid points defined around target proteins for calculating MIFs(func, X) are therefore defined by laboratory coordinates. Therefore, for the MIF(func, X) calculation, the grid points in the laboratory coordinates (*o-xyz*) (Figure 3b) are transformed to molecular coordinates (*o-uvw*) (Figure 3b) according to the orientations of amino acid residues, respectively. Following the transformation, the MIF(func, X) energies are calculated by adding the obtained *u*, *v*, and *w* components to the approximation function *E*_*X*_(r). The detailed procedure is described in the Supporting Information.

Considering steric repulsion from proteins, the MIF(func, X) energies of the grid points within 2 Å from the protein atoms were set to 0. When two or more amino acid residues that contribute to the MIF(func, X) value exist, the MIF(func, X) value arising from the residue that exhibits the highest contribution was adopted.

### Computational Details

All QM calculations were performed using the Gaussian16 program.^50^ The pseudopotentials were obtained from the Basis Set Exchange Web site (https://www.basissetexchange.org/).^51,52^ The s-, p-, d-, f-, g-, h-, i-, and j-type CGFs were adopted for the Gaussian expansion of the approximation function E_X_(r). In the calculations, CGFs with four different exponent values, αs were defined for s, p, d, f, g, h, i, and j type functions. Additionally, 1 s-, 3 p-, 6 d-, 10 f-, 15 g-, 21 h-, 28 i-, and 36 j- type CGFs were included. Thus, a total of 480 (4 × 120) CGFs were used in this study. The exponents αs of the Gaussian functions are listed in Table 1. Fitting calculations were performed using our in-house code, programmed in Fortran. The program was developed by expanding a previously proposed density fitting method.^40,41^ The BLAS-LAPAC library was used for matrix calculations (https://www.netlib.org/lapack/). The molecules were depicted using the Visual Molecular Dynamics (VMD) program.^53^

**Table 1:** Values of Exponent α of the Cartesian Gaussian Functions (CGFs)

	CGF-1^a^	CGF-2^a^	CGF-3^a^	CGF-4^a^
*s*	0.03	0.08	0.13	0.18
*p*	0.03	0.08	0.13	0.18
*d*	0.056	0.106	0.156	0.206
*f*	0.083	0.133	0.183	0.233
*g*	0.111	0.161	0.211	0.261
*h*	0.139	0.189	0.239	0.289
*i*	0.167	0.217	0.267	0.317
*j*	0.194	0.244	0.294	0.344

a CGFs with four different values of exponent α defined for *s*, *p*, *d*, *f*, *g*, *h*, *i*, and *j* type functions.

## Results and Discussion

### Fitting Calculations of the Approximation Functions of MIFs

**Figure 4:**
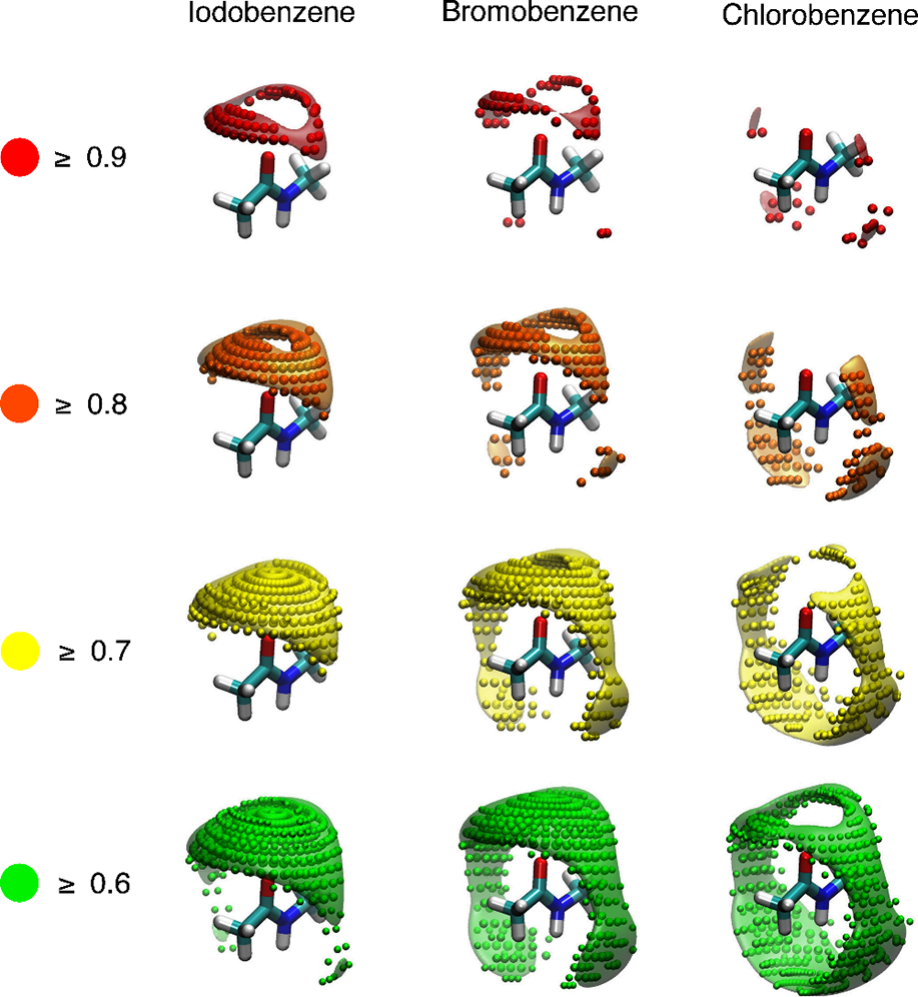
MIFs(QM, X) of *N*-methylacetamide calculated using iodobenzene (left), bromobenzene (center), and chlorobenzene (right) probes. The MIF energy values were normalized by the most stable energy values. MIF(QM, Cl), MIF(QM, Br), and MIF(QM, I) values of 1.0 correspond to interaction energies of −1.89, −2.35, and −3.92 kcal/mol, respectively. 3D alternations of MIFs(QM, X) are depicted by spheres, color coded according to the MIF energy, while those of MIFs(func, X) are described by surface representation with according to the same color key.

According to Kozach et al., the ωB97X-D density functional and aug-cc-pVTZ basis set are suitable for describing halogen bonding.^54^ Additionally, the use of pseudopotentials for bromine and iodine atoms, and counterpoise corrections are required.^54^ To examine the validity of the ωB97X-D/aug-cc-pVDZ-PP calculations adopted in the system, the MIF(QM, Br) for *N*-methylacetamide and bromobenzene was calculated using ωB97X-D/aug-cc-pVTZ-PP and ωB97X-D/aug-cc-pVDZ-PP. The coefficient of determination (r^2^) between the MIF(QM, Br) energies calculated using ωB97X-D/aug-cc-pVTZ-PP and those obtained using ωB97X-D/aug-cc-pVDZ-PP was 1.0 (Figure S1). This suggests that the ωB97X-D/aug-cc-pVDZ-PP calculations accurately reproduce the results obtained using ωB97X-D/aug-cc-pVTZ-PP for the present system. The MIFs(QM, X) for *N*-methylacetamide and the iodobenzene or bromobenzene probe calculated using ωB97X-D/aug-cc-pVDZ-PP are shown in Figure 4. The MIFs(QM, X) are depicted using heat maps (small colored spheres), which represent alternations in the normalized interaction energies of the halogen bonds around the *N*-methylacetamide molecule. Each halogen species gave rise to a distinct heat map. In the cases of iodobenzene and bromobenzene, the most stable halogen-bond-formable regions (≥0.9) are located around the carbonyl oxygen above the equator (Θ_2_ > 90). Bond formation in this region is indicative of typical C-X/O halogen bonds. The halogen bonds formed around the pole (Θ_2_ ≈ 180°) are relatively weak (≈0.6–0.7). The obtained MIFs(QM, X) are in good agreement with the spherical scores for *N*-methylacetamide reported by Zimmermann et al.^42^ The interaction energies of the most stable points are −3.92 and −2.35 kcal/mol for the iodobenzene and bromobenzene probes, respectively. Thus, a MIF(QM, I) and MIF(QM, Br) value of 1.0 corresponds to interaction energies of −3.92 and −2.35 kcal/mol, respectively. In the case of the chlorobenzene system, the ωB97X-D/aug-cc-pVDZ calculation generated a MIF(QM, Cl) with values that are globally weaker than those of the spherical score^42^ (Figure S2). On the contrary, the MP2/aug-cc-pVDZ calculation delivered a MIF(QM, Cl) with features closely resembling those of the spherical score.^42^ The MP2/aug-cc-pVDZ method was therefore adopted for the chlorobenzene system. In the MIF(QM, Cl), the most stable halogen-bond-formable regions (≥0.9) are mainly situated around the equator or below it (Θ_2_ ≤ 90). This translates to halogen bond formation between the C–Cl group of the probe and π-electron of the amide portion. These tendencies were also observed in the calculation results reported by Zimmermann et al.^42^ In addition, a MIF(QM, Cl) value of 1.0 corresponds to an interaction energy of −1.89 kcal/mol.

The MIFs(func, X) calculated using the approximation functions *E*_*X*_(r), obtained from fitting calculations, are depicted in Figure 4 as colored surfaces. For all tested halobenzenes, the MIFs(func, X) successfully reproduced the MIFs(QM, X). The coefficients of determination (r^2^) for the iodobenzene, bromobenzene, and chlorobenzene probes were 0.96, 0.93, and 0.92, respectively (Figure S1), indicating that the calculated functions *E*_*X*_(r) are good approximations of MIFs(QM, X). Additionally, Figure 4 shows that MIFs(QM, X) and MIFs(func, X) depend significantly on the Ψ parameter. This suggests that parameters *d*, Θ_1_ and Θ_2_ cannot completely describe 3D halogen bond strength alternations. Approximation functions *E*_*X*_(r) that describe the dependence of halogen bond strength on Ψ would therefore be convenient for characterizing halogen bonds. The MIFs(func, X) depicted in Figure 4 are shown in more detail in Figures S3, S4, and S5.

### MIF Mapping on the Ligand Binding Sites of Proteins

Protein MIFs(func, X) were calculated using the approximation functions *E*_*X*_(**r**) according to the procedure described in the Methods section. The method was applied to the Cyclin-dependent protein kinase 2(CDK2)/ligand (TBS) system (PDB ID: 1P5E) and the results are shown in Figure S6.^55^ 150 × 150 × 150 = 3,375,000 grid points with intervals of 0.5 Å were defined around CDK2. The mapping calculation was completed within 190 s when a single processor was used (Intel(R) Xeon(R) Gold 6248 CPU @ 2.50 GHz). As shown in Figure S6, MIF mapping of the entire protein structure was achieved within a realistic computation time.

**Figure 5:**
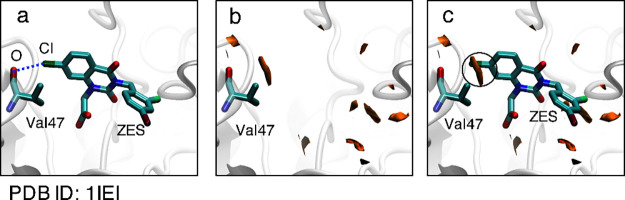
(a) Ligand binding site of the aldose reductase (AR)/ligand (ZES) complex structure (PDB ID: 1IEI); the dashed line represents the formed O/Cl halogen bond. (b) The calculated MIF(func, Cl) for the ligand binding site of the AR structure. Halogen-bond-formable areas with halogen bond strengths of 0.8 or higher are shown as orange surfaces. (c) The superposition of the AR/ZES complex and calculated MIF(func, Cl).

**Figure 6:**
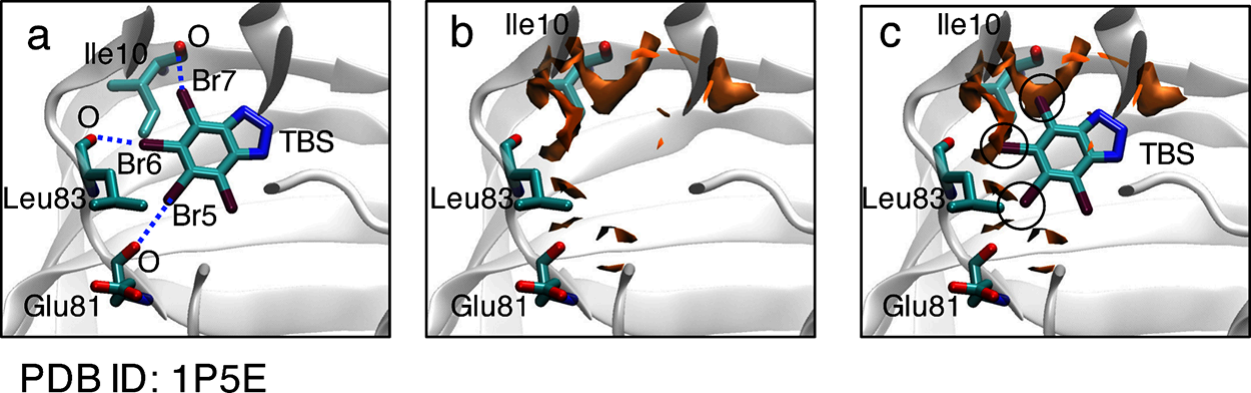
(a) Ligand binding site of the cyclin-dependent kinase 2 (CDK2)/ligand (TBS) complex structure (PDB ID: 1P5E). The dashed line represent the three formed O/Br halogen bonds. (b) The calculated MIF(func, Br) for the ligand binding site of the CDK2 structure. Halogen-bond-formable areas with halogen bond strengths of 0.8 or higher are shown as orange surfaces. (c) The superposition of the CDK2/TBS complex and calculated MIF(func, Br).

**Figure 7:**
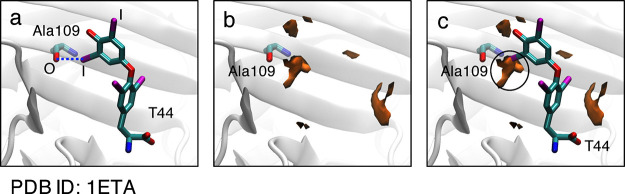
(a) Ligand binding site of the transthyretin (TTR)/ligand (T44) complex structure (PDB ID: 1ETA). The dashed line represents the formed O/I halogen bond. (b) The calculated MIF(func, I) for the ligand binding site of the TTR structure. Halogen-bond-formable areas with halogen bond strengths of 0.8 or higher are shown as orange surfaces. (c) The superposition of the TTR/T44 complex and calculated MIF(func, I).

MIFs(func, X) around ligand binding sites are especially meaningful for SBDD. The MIFs(func, X) of the ligand binding sites of aldose reductase (AR) (PDB ID: 1IEI),^56^ CDK2 (PDB ID: 1P5E),^55^ and Transthyretin (TTR) (PDB ID: 1ETA)^57^ are shown in Figures 5, 6, and 7, respectively. The X-ray crystal structure of the AR/ligand (ZES) complex indicated that a C–O/Cl halogen bond had formed between the carbonyl oxygen of the Val47 of AR and the chloride atom of the ligand molecule (ZES) (Figure 5a).^56^ The parameters of the halogen bond are listed in Table 2. Figure 5b shows the MIF(func, Cl) calculated for the AR structure without the ligand molecules. Although MIF mapping calculations do not require ligand–molecule information, in the present calculations, the Cartesian coordinates of the ligand molecules were used to determine the ligand-binding sites. For simplicity, MIF calculations were performed for regions within 4 Å from the ligand molecules. The orange-colored areas indicate halogen-bond-formable areas with halogen bond strengths of 0.8 or higher (Figure 5b). The AR/ligand complex structure and the MIF(func, Cl) of the ligand binding site of AR are superimposed in Figure 5c. The circle in Figure 5c shows the MIF(func, Cl) cloud (≥0.8) covering the chloride atom of the ligand molecule. The positional overlap of the MIF(func, Cl) cloud and the chloride atom, which is located at a favorable site for halogen bond formation, implies that the halogen bond formable areas were appropriately defined by the MIF(func, Cl). The calculated halogen bond strength at the chloride atom was 0.85 (Table 2).

**Table 2:** Relevant Parameters of C=O/X Halogen Bonds Formed in the AR/ZES(X=Cl), CDK2/TBS(X=Br), TTR/T44(X=I), and HSA/T44(X=I) Complex Systems^a^

proteins	PDB ID	chain	res	ligand	*d*	Θ_1_	Θ_2_	Ψ	strength	reference
AR	1IEI	A	Val47	ZES	3.15	156.94	98.87	–86.63	0.85	(56)
CDK2	1P5E	A	Leu83	TBS	2.90	164.70	131.98	137.32	0.59	(55)
CDK2	1P5E	A	Ile10	TBS	3.24	144.08	110.05	–85.12	0.96	(55)
CDK2	1P5E	A	Glu81	TBS	3.01	168.55	154.87	–41.29	0.67	(55)
TTR	1ETA	1	Ala109	T44	3.13	149.45	106.13	–89.20	0.93	(57)
HSA	1HK4	A	Asn429	T44	3.21	132.53	113.58	–152.51	0.00	(90)

a AR: Aldose reductase; CDK2: cyclin-dependent protein kinase 2; TTR: transthyretin; HSA: human serum albumin.

In the case of the CDK2/ligand (TBS) system (PDB ID: 1P5E), three halogen bonds are formed: the carbonyl oxygens of Ile10, Glu81, and Leu83 interact with Br7, Br5, and Br6 of the TBS molecule, respectively (Figure 6a and Table 2).^55^Figure 6b shows the calculated MIF(func, Br) around the ligand binding site. The CDK2/TBS complex and MIF(func, Br) are superimposed in Figure 6c.

The MIF(func, Br) appropriately captured the position of the Br7 bromine atom, which forms a halogen bond with Ile10. The halogen bond strength at the bromine position is 0.96 (Table 2). This suggests that the bromine atom is located in the optimal position for the formation of a halogen bond with Ile10. On the contrary, the MIF(func, Br) clouds around bromine atoms Br5 and Br6, which form halogen bonds with Glu81 and Leu83, respectively, are sparse. The halogen bond strengths at the Br5 and Br6 positions are 0.67 and 0.59 (Table 2), respectively. However, MIF(func, Br) clouds with bond strengths of 0.8 or higher are formed close to these bromine atoms. Considering thermal fluctuation, these bromine atoms can potentially form favorable halogen bonds at additional locations on CDK2.

The X-ray crystal structure of the TTR/ligand (T44) complex (PDB ID: 1ETA) suggests that a halogen bond is formed between the carbonyl oxygen of Ala109 and an iodine atom of the ligand (Figure 7a).^57^Figure 7b shows the calculated MIF(func, I) for the ligand binding site of TTR. Figure 7c displays the TTR/T44 complex superimposed on the MIF(func, I), wherein it is evident that the MIF(func, I) appropriately described the halogen-bond-formable area. The halogen bond strength at the position of the iodine atom is 0.93 (Table 2).

### Examination of MIFs(func, X) for 68 Halogen Bonds Formed in Protein/Ligand Complexes

Auffinger et al. described halogen bond formation in protein/ligand complexes based on analyses of X-ray crystal structures registered in the PDB.^28^ The bonds proposed by Auffinger et al. cannot be unequivocally classified as halogen bonds as they were identified based solely on the geometries observed in X-ray crystal structures. Nonetheless, they are widely accepted as typical examples, and regarded as appropriate data sets for considering halogen bonds. Therefore, the validity of the MIF(func, X) calculation was evaluated by applying the MIF calculations to Auffinger’s data set.^28^ The strengths of the halogen bonds were determined using the MIF(func, X) calculation and compared to reported bond positions and geometries.

As mentioned, the focus of the study was the description of halogen bonds that form between the carbonyl oxygen atoms of protein main chains and the halogen atoms of ligands. The halogen bonds formed between the side chains and ligands were excluded from the analysis. Furthermore, only ligand molecules with halobenzene moieties were considered, while others, such as alkyl halides, were excluded. In the present analysis, halogen bond strengths were evaluated at the halogen atoms of the donors for 23 C–Cl/O,^56,58−71^ 18 C–Br/O,^55,72−79^ and 27 C–I/O^57,80−91^ bonds using the MIF(func, X) calculation.

The calculation results are presented in Table S1–S3 in the Supporting Information. As described in the above section, the MIF(func, X) energies were normalized using the most stable energy. Thus, the regions or points with positive halogen bond strength values are halogen-bond-formable. A halogen bond strength of zero suggests that the region or point is unfavorable for halogen bond formation. Of the 68 halogen bonds analyzed, the calculated strengths of 64 were positive, while those of four were zero (Tables S1–S3). This suggests that the halogen atoms of 64 halogen bond donors are located in the predicted halogen-bond-formable areas. If we assume the real existence of all the halogen bonds proposed by Auffinger et al., it can be interpreted that the calculated MIFs(func, X) appropriately describe the halogen-bond-formable areas for a large majority of cases.

**Figure 8:**
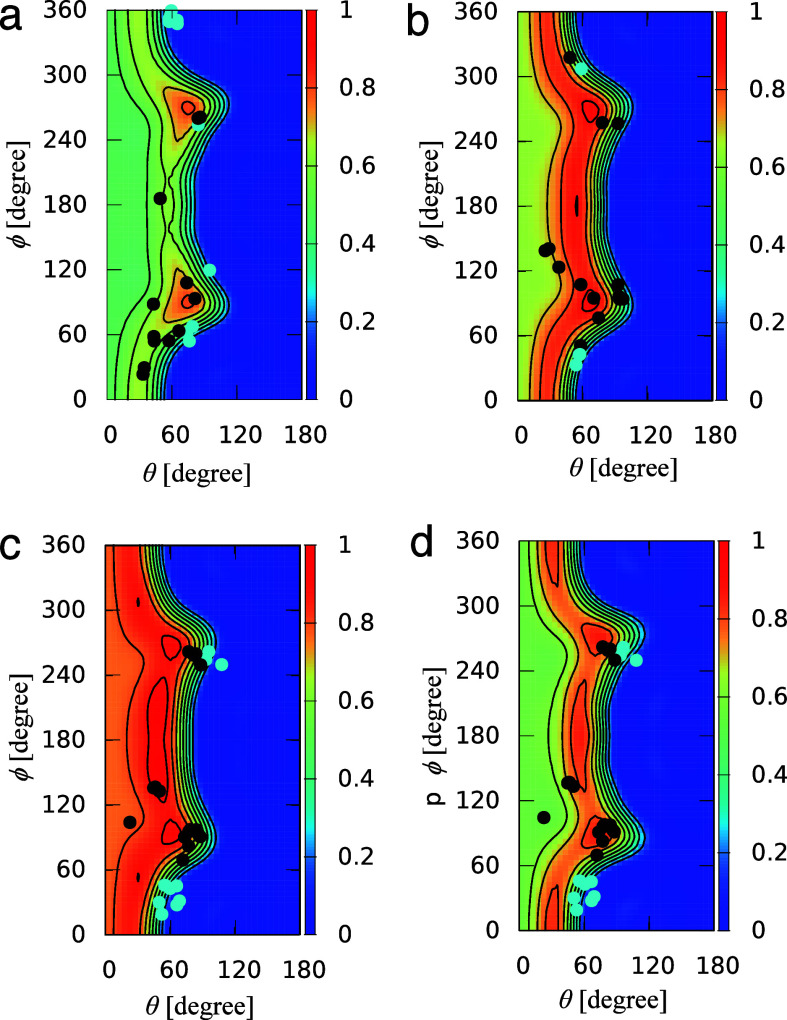
MIF(func, X) dependence on θ and φ for (a) MIF(func, Cl), (b) MIF(func, Br), (c and d) MIF(func, I). The 2D maps describe the MIF(func, X) energies when the *d* values are 3.0 Å for (a), (b), and (c), and 3.5 Å for (d). The θ (=180 – Θ_2_) and φ (=180 + Ψ) values of each halogen bond investigated here were plotted on the 2D maps. The θ and φ values of the halogen bonds with strengths of 0.5 or higher are plotted in black. The θ and φ values of the halogen bonds with strengths lower than 0.5 are plotted in cyan.

The relationship between the halogen bond strength and the parameters *d*, Θ_1_, Θ_2_, and Ψ was investigated. It is well-known that the halogen bond strength depends on parameters *d* and Θ_1_. In protein/ligand systems, the average values of *d* for chlorinated, brominated, and iodinated compounds are 3.06, 3.15, and 3.24 Å, respectively.^28^ The halogen bond strength gradually weakens with an increase or decrease in *d*, being the optimal length. Furthermore, it reaches a maximum when Θ_1_ = 180°.^24^ The straightforward dependence of the halogen bond strength on *d* and Θ_1_ allows for a high degree of predictability. In the halogen bonds considered in the present analyses, the values of *d* ranged from 2.8 to 3.5, and those of Θ_1_ were generally larger than 120° (Table S1–S3). On the contrary, the relatively complex relations between the halogen bond strength and parameters Θ_2_ and Ψ have not been discussed in previous studies. Figure 8 shows the two-dimensional (2D) MIF(func, X) maps, illustrating the dependence of the halogen bond strength on θ (= 180 – Θ_2_) and φ (= 180 + Ψ). The 2D maps show the MIF(func, X) values when *d* = 3.0 or 3.5 Å. The θ and φ values for each halogen bond are also plotted on the 2D maps and correspond to the Θ_2_ and Ψ values, respectively, listed in Tables S1–S3. The 2D maps are useful for understanding the relationship between the halogen bond strength and parameters Θ_2_ and Ψ. In Figure 8, the θ and φ values of halogen bonds with strengths of 0.5 or larger are located within the halogen-bond-formable areas, that is, areas with a positive strength value (black points). The 2D plots show that the θ and φ values of almost all the halogen bonds with strengths below 0.5, including the four with zero strength, are scattered around the borderlines (cyan points). This implies that, in the case of the halogen bonds with strengths below 0.5, the values of *d* and Θ_1_ parameters are favorable, while the values of Θ_2_ and Ψ are on the borderline between the favorable and unfavorable regions, from the MIF point of view. Furthermore, halogen bond formation was not observed in the unfavorable area far from the borderlines. The results suggest that the halogen-bond-formable areas predicted by the MIF(func, X), in turn obtained by applying QM calculations using a model molecule, are largely in accord with the bonds observed in the X-ray crystal structures of protein/ligand systems.

**Figure 9:**
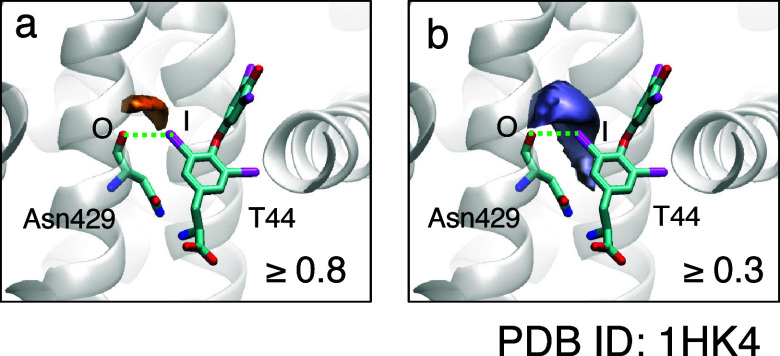
Ligand binding site of the human serum albumin (HSA)/ligand (T44) complex structure (PDB ID: 1HK4). The calculated MIF(func, I) are shown by surface representations. (a) Halogen-bond-formable area with bond strengths of 0.8 or higher. (b) Halogen-bond-formable area with bond strengths of 0.3 or higher. The C–I/O halogen bond formed between the carbonyl oxygen of Asn429 and an iodine atom of T44 is represented by the dashed line. For simplicity, only the effect of Asn429 was evaluated in the MIF(func, I) calculation.

Figure 8 suggests that some halogen bonds are formed in the boundary region between the halogen-bond-formable and nonformable areas. Herein, the validity of this prediction was evaluated using the MIFs(func, X) of protein surfaces. Figure 9 shows the binding site of the human serum albumin (HSA)/ligand (T44) complex (PDB ID: 1HK4);^90^ the calculated MIFs(func, I) are depicted by surface representations. Additionally, the more detailed figures are shown in Figure S7. Figure 9a shows the C–O/I halogen bond formed between the carbonyl oxygen of Asn429 and the iodine atom of the ligand in the complex. For simplicity, only the effect of Asn429 was evaluated in the MIF(func, I) calculation. Figure 9a shows that the MIF(func, I) cloud (≥0.8) does not cover the iodine atom of the ligand, and the halogen bond strength on the iodine atom is 0.00 (Table 2). However, the locus predicted to be favorable for halogen bond formation (≥0.8) is close to the iodine atom (Figure 9a). The distance between the favorable site and the iodine atom is shorter than the C–I bond length. Moreover, Figures 9b show that the iodine atom is located close to the edge of the cloud defining the halogen-bond-formable area with halogen bond strength of 0.3 or higher. This result supports the formation of a halogen bond in the boundary region between the halogen-bond-favorable and nonfavorable areas. Similar results were observed for the enoyl reductase (ENR)/ligand (TCL)(PDB ID: 1C14), HSA/ligand (T44) (PDB ID: 1HK5), and transthyretin (TTR)/ligand (T44) (PDB ID: 1ICT) complexes (Figures S8–S10).

Precise prediction of the halogen bond strength in the boundary region is technically difficult in the current status because of the steep slope around the boundary region. As an alternative, the underestimated halogen bonds can be retrieved using switching functions or cubic splines. However, in practical applications such as drug design, typically the aim is to design a molecule that forms a favorable halogen bond. Thus, in practice, it is not necessary to focus on halogen bonds in the boundary region.

The calculated halogen bond strengths for 64 halogen bonds formed in various protein/ligand complexes were positive. This suggests that the halogen atoms of the halogen bond donors in the complexes are located in the predicted halogen bond-formable areas. The agreement between the calculation results and experimental crystal structures suggests the validity of the MIF calculations. Additionally, the halogen bonds formed in the boundary region between the halogen-bond-favorable and-unfavorable areas generally exhibit weak strength in MIF(func, X) calculations. Overall, it can be concluded that the MIF(func, X) calculations generated reasonable results.

## Conclusions

A method for obtaining the approximation functions of QM-level MIFs using small model molecules is proposed. We applied this method to calculate the approximation functions of MIFs (QM, X) of *N*-methylacetamide with chlorobenzene, bromobenzene, and iodobenzene probes. The obtained functions, *E*_*X*_(**r**), were confirmed to be good approximations for QM-level MIFs. Moreover, we proposed a method for mapping MIFs(func, X) on protein surfaces using the approximation functions within a realistic computation time. To examine the proposed method, we calculated MIFs(func, X) in the ligand binding sites of proteins where halogen bonds are formed. The developed method appropriately reproduced the halogen-bond-formable areas around the ligand-binding sites of proteins. Finally, we evaluated the bond strengths of 68 halogen bonds formed in protein/ligand complexes using MIF(func, X) calculations. Positive strength values were calculated for 64 halogen bonds. In addition, the halogen bonds formed in the boundary region between the halogen-bond-favorable and-unfavorable areas generally exhibit weak strength in MIF(func, X) calculations. The results suggest the validity of the MIF calculation for the protein surfaces proposed herein. The present methodology constitutes a useful tool for in silico SBDD.

## Supporting Information Available

Figure S1. Correlation between the MIF(QM, Br) energies of *N*-methylacetamide determined using ωB97X-D/aug-cc-pVDZ-PP and ωB97X-D/aug-cc-pVTZ-PP calculations. Correlation between the MIF(QM, X) energies calculated using ωB97X-D/aug-cc-pVDZ-PP and predicted by the approximated functions *E*_*X*_(**r**). Figure S2. MIF(QM, Cl) of *N*-methylacetamide estimated by ωB97X-D/aug-cc-pVDZ calculation. Figures S3–S5. MIF(QM, X), MIF(func, X), and their superposition. Figure S6. Calculated MIF(func, Br) for an entire CDK2 structure (PDB ID: 1P5E). Table S1. Relevant parameters of C=O/Cl halogen bonds formed in various protein/ligand systems. Table S2. Relevant parameters of C=O/Br halogen bonds formed in various protein/ligand systems. Table S3. Relevant parameters of C=O/I halogen bonds formed in various protein/ligand systems. Figure S7. Ligand binding site of the HSA/ligand (T44) complex structure (PDB ID: 1HK4) with the calculated MIF(func, I). Figure S8. Ligand binding site of the ENR/ligand (TCL) complex structure (PDB ID: 1C14) with the calculated MIF(func, Cl). Figure S9. Ligand binding site of the HSA/ligand (T44) complex structure (PDB ID: 1HK5) with the calculated MIF(func, I). Figure S10. Ligand binding site of the TTR/ligand (T44) complex structure (PDB ID: 1ICT) with the calculated MIF(func, I) (PDF)
Input file of Gaussian16 program: *N*-Methylacetamide-chlorobenzene-g16 (ZIP)
Input file of Gaussian16 program: *N*-Methylacetamide-bromobenzene-g16 (ZIP)
Input file of Gaussian16 program: *N*-Methylacetamide-iodobenzene-g16 (ZIP)
Obtained MIF(QM, X) and parameters of MIF(func, X): MIF_QM_FUNC (XLSX)
Water deleted PDB format file used for the MIF(func, X) mapping: 1p5e-ba1-chain-a-nowater (PDB)
Water deleted PDB format file used for the MIF(func, X) mapping: 1iei-ba1-nowater (PDB)
Water deleted PDB format file used for the MIF(func, X) mapping: 1p5e-ba1-nowater (PDB)
Water deleted PDB format file used for the MIF(func, X) mapping: 1eta-nowater (PDB)
PDB format file used to calculate halogen bond strength (the positions of the halogen atoms of the respective donors are contained in the same file): struct_xbond_strength_cl (TXT)
PDB format file used to calculate halogen bond strength (the positions of the halogen atoms of the respective donors are contained in the same file): struct_xbond_strength_br (TXT)
PDB format file used to calculate halogen bond strength (the positions of the halogen atoms of the respective donors are contained in the same file): struct-xbond-strength-i (TXT)

## Supplementary Material

ci4c00896_si_005.xlsx

ci4c00896_si_006.pdb

ci4c00896_si_007.pdb

ci4c00896_si_008.pdb

ci4c00896_si_009.pdb
