# Supplementary material for: Molecular Interaction Fields Describing Halogen Bond Formable Areas on Protein Surfaces
Source: J Chem Inf Model. 2024 Jul 16;64(15):6003–13. doi: 10.1021/acs.jcim.4c00896 (PMC11323840; doi:10.1021/acs.jcim.4c00896)
Supplement: Supplementary file 1 — ci4c00896_si_001.pdf [file ci4c00896_si_001.pdf]

Supporting Information

Molecular Interaction Fields Describing Halogen Bond Formable Areas on Protein Surfaces

Daichi Hayakawa, Yurie Watanabe, and Hiroaki Gouda*.*

Division of biophysical chemistry, Department of pharmaceutical sciences, Graduate School of Pharmacy, Showa University, 1-5-8 Hatanodai, Shinagawa-ku, Tokyo, 142-8555, Japan.

*d-hayakawa@pharm.showa-u.ac.jp
*godah@pharm.showa-u.ac.jp

The 12-step calculation procedure of the protein MIF(func, X)………………….2
Figure S1…………………………………………………………………….3
Figure S2…………………………………………………………………….4
Figure S3…………………………………………………………………….5
Figure S4…………………………………………………………………….6
Figure S5…………………………………………………………………….7
Figure S6…………………………………………………………………….8
Table S1…………………………………………………………………...9
Table S2…………………………………………………………………..11
Table S3…………………………………………………………………..12
Figure S7…………………………………………………………………....14
Figure S8…………………………………………………………………....15
Figure S9…………………………………………………………………....16
Figure S10……………………………………………………………….…..17

The 12-step calculation procedure of the protein MIF(func, X)

In the MIF(func, X) calculations, coordinate transformations are required as mentioned in the main text. Considering the coordinate transformations, the protein MIFs(func, X) were calculated using the following 12-step procedure.

(i) Grid points ($\mathbf{r}_{xyz}$) are defined around the target protein. The grid point coordinates r_{xyz} are represented in laboratory coordinates (*o-xyz*).

(ii) One grid point is selected.

(iii) A single amino acid residue is selected from the target protein. The coordinate of the grid point r_{xyz} is translated to $r_{x'y'z'}$ such that the carbonyl oxygen of the target amino acid becomes the origin of the coordinate. The translated coordinate is denoted as *o-x'y'z'*.

(iv) With the carbonyl oxygen defining as the origin of the molecular coordinate (*o-uvw*), the *w*-axis is defined along the C-O axis (Figure 3b).

(v) The *u*-axis is defined on the C-O-Cα plane perpendicular to the *w*-axis (Figure 3b).

(vi) The *v*-axis is perpendicular to *v*- and *w*-axes. (Figure 3b)

(vii) The Euler angles of *o-uvw* with respect to *o-x'y'z'* are calculated.

(viii) The rotation matrix **R** is determined based on the calculated Euler angles.

(ix) The coordinate of the grid point $\mathbf{r}_{x'y'z'}$ is transformed to r_{uvw} using the matrix **R**.

(x) The components of the coordinate $\mathbf{r}_{uvw}$ are added to the approximation function $E_X(\mathbf{r})$, from which the MIF(func, X) energy value at the grid point is obtained.

(xi) Steps (iii)–(x) are repeated for all the amino acids contained in the target protein.

(xii) Steps (ii)–(xi) are repeated for all grid points defined in step (i).

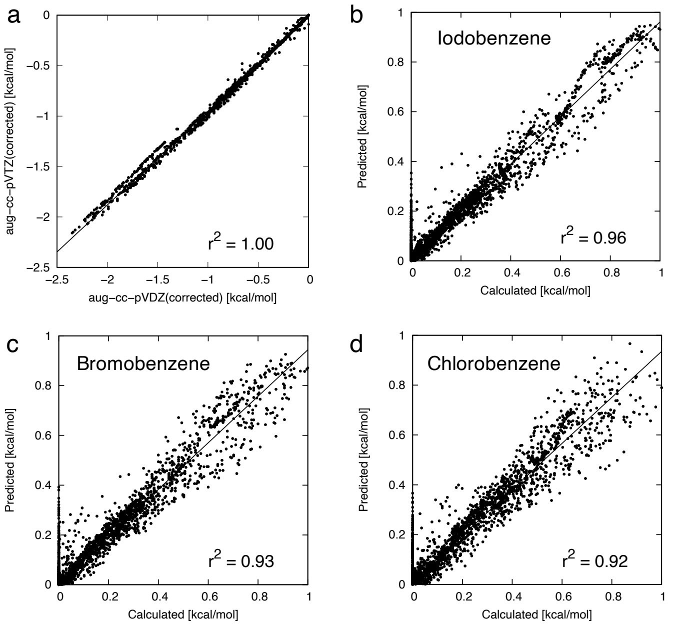

Figure S1. a) Correlation between the MIF(QM, Br) energies of N-methylacetamide determined using the ωB97X-D/aug-cc-pVDZ-PP and ωB97X-D/aug-cc-pVTZ-PP calculations. b) – d) The correlations between the MIF(QM, X) energies calculated using ωB97X-D/aug-cc-pVDZ-PP and predicted by the approximated functions $E_X(\mathbf{r})$ for X = I, Br, Cl, respectively.

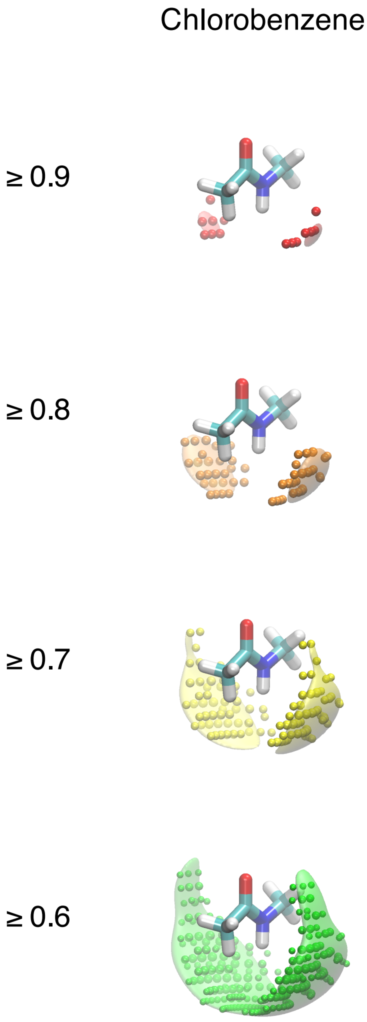

Figure S2. MIF(QM, Cl) of N-methylacetamide estimated by the ωB97X-D/aug-cc-pVDZ calculation. The MIF energy values were normalized by the most stable energy values. A MIF(QM, Cl) value of 1.0 corresponds to the interaction energy of -1.69 kcal/mol. Three-dimensional alternations of MIF(QM, Cl) are represented by spheres color coded according to the MIF energy. Those of MIF(func, Cl) are described by surface representations with the same color key..

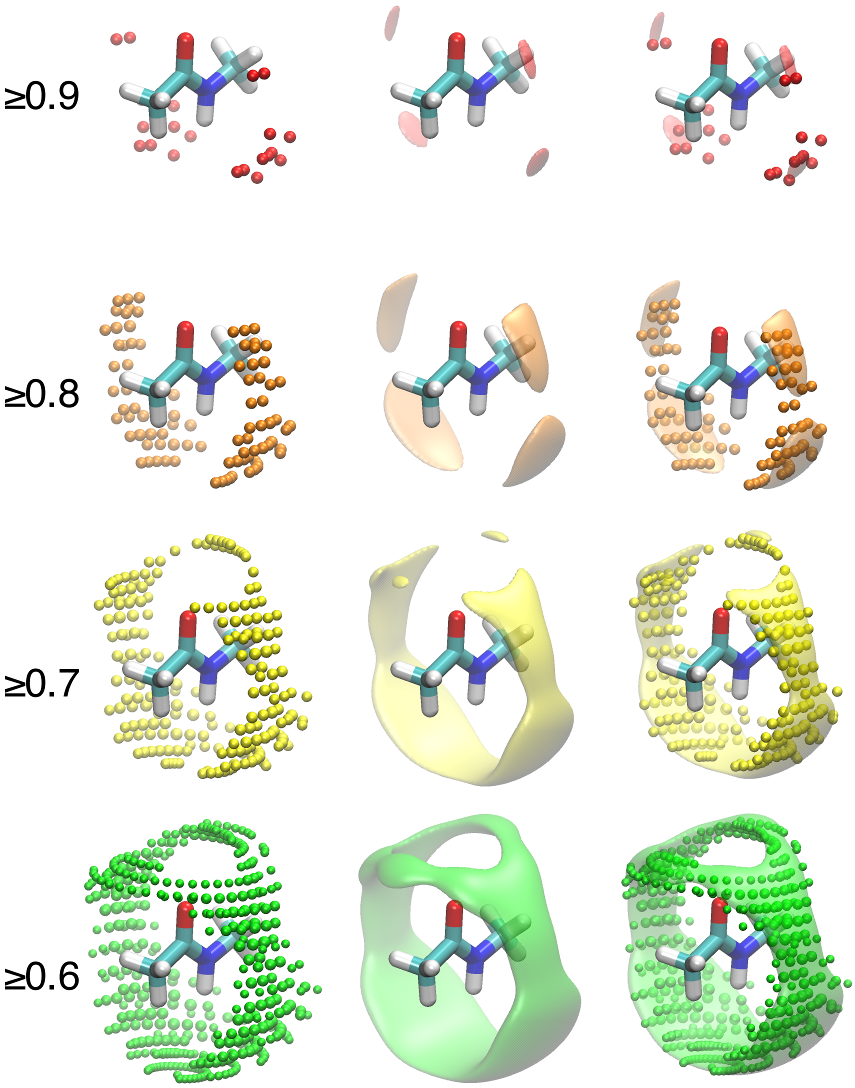

Figure S3. MIF(QM, Cl) of N-methylacetamide estimated by the MP2/aug-cc-pVDZ calculation (left). MIF(func, Cl) obtained by the fitting calculation for MIF(QM, Cl) (center). Superpositions of MIF(QM, Cl) and MIF(func, Cl) (right). MIF(QM, Cl) and MIF(func, Cl) are described by colored spheres and surfaces, respectively. The first, second, third, and forth lines correspond to the MIFs represented with thresholds of 0.9, 0.8, 0.7, or 06, respectively.

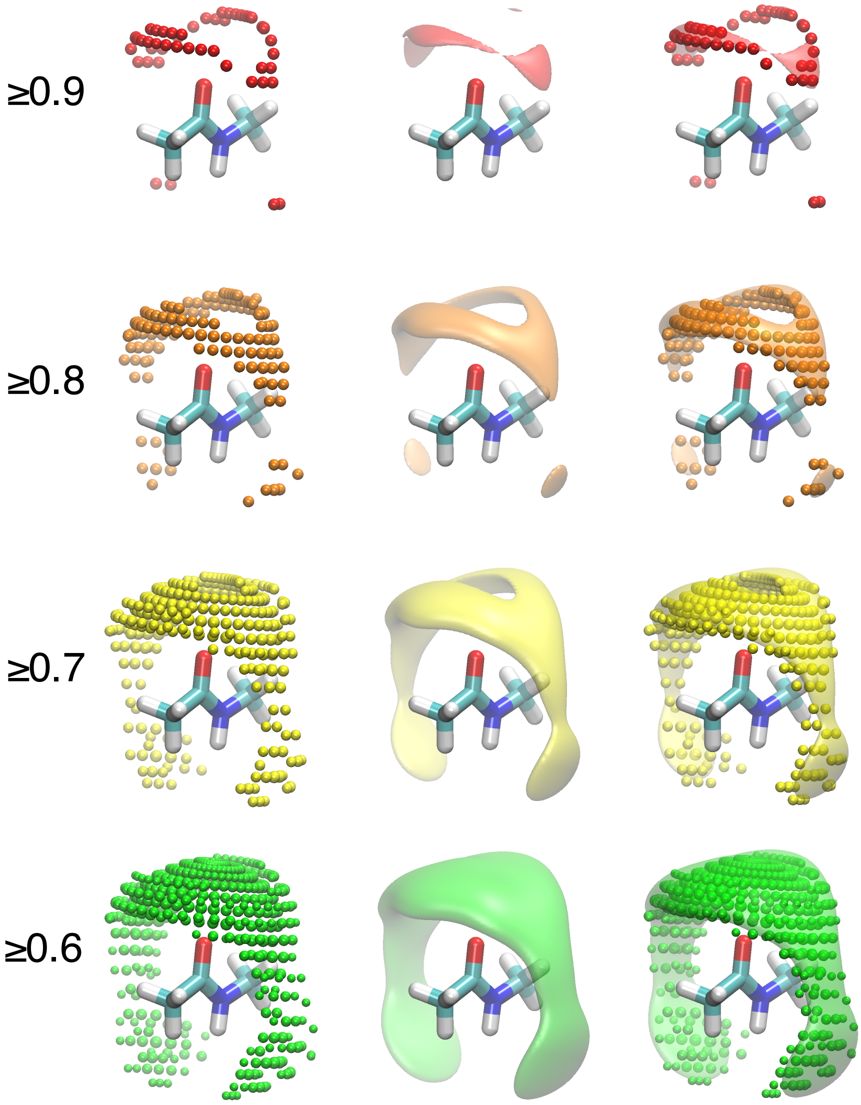

Figure S4. MIF(QM, Br) of N-methylacetamide estimated by the ωB97X-D/aug-cc-pVDZ-PP calculation (left). MIF(func, Br) obtained by the fitting calculation for MIF(QM, Br)(center). Superposition of MIF(QM, Br) and MIF(func, Br) (right). MIF(QM, Br) and MIF(func, Br) are described by colored spheres and surfaces, respectively. The first, second, third, and forth lines correspond to the MIFs represented with thresholds of 0.9, 0.8, 0.7, or 06, respectively.

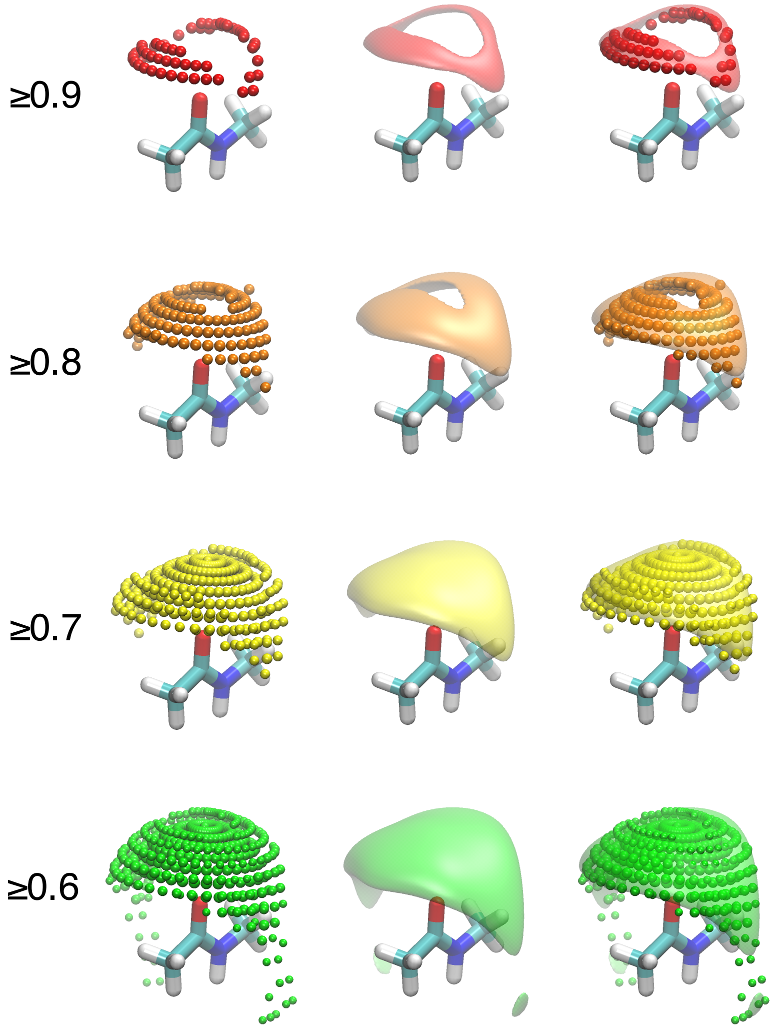

Figure S5. MIF(QM, I) of N-methylacetamide estimated by the ωB97X-D/aug-cc-pVDZ-PP calculation (left). MIF(func, I) obtained by the fitting calculation for MIF(QM, I) (center). Superposition of MIF(QM, I) and MIF(func, I) (right). MIF(QM, I) and MIF(func, I) are described by colored spheres and surfaces, respectively. The first, second, third, and forth lines correspond to the MIFs represented with thresholds of 0.9, 0.8, 0.7, or 06, respectively.

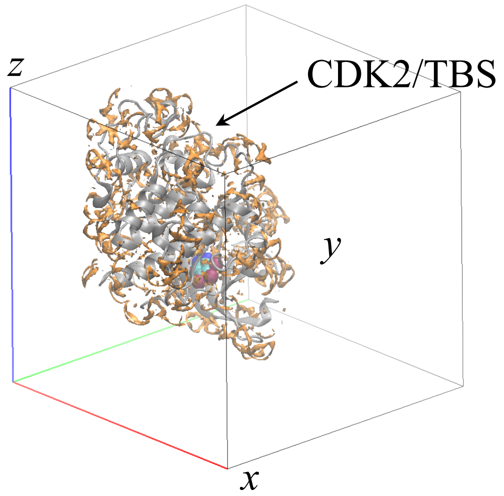

Figure S6. The calculated MIF(func, Br) for an entire CDK2 structure (PDB ID: 1P5E).

Table S1. Relevant parameters of C=O/Cl halogen bonds formed in various protein/ligand systems.

Proteins	PDB ID	Chain	Res	Ligand	d	Θ1	Θ2	Ψ	strength	reference
**ENR**	1C14	A	Ala95	TCL	3.25	172.86	115.17	170.72	0.00	59
**ENR**	1C14	B	Ala1095	TCL	3.46	166.93	115.05	167.58	0.01	59
**3HNR**	1G0N		Met215	PHH	3.03	163.66	85.18	-60.32	0.20	60
**hEST**	1G3M	B	Ile246	PCQ	3.14	146.87	122.85	-125.69	0.56	65
**AR**	1IEI		Val47	ZES	3.15	156.94	98.87	-86.63	0.85	56
**PfDHFR-TS**	1J3J	A	Asn108	CP6	3.06	174.78	103.91	-125.86	0.20	68
**PfDHFR-TS**	1J3J	B	Asn108	CP6	3.22	168.45	113.77	-116.42	0.63	68
**DNA Gyrase**	1KZN		Gly77	CBN	3.20	143.51	106.44	-72.00	0.79	63
**hLGP**	1L7X	A	Arg60	700	3.26	121.29	136.47	-125.78	0.65	62
**hLGP**	1L7X	B	Arg60	700	3.27	117.64	136.83	-121.71	0.66	62
**F16BPase**	1LEV	F	Val160	CLI	3.10	160.68	137.18	-91.67	0.58	67
**DHBD**	1LKD		Ser255	CB6	3.03	164.31	130.93	5.88	0.60	61
**DHFR**	1M78	A	Ile112	CLZ	3.26	129.63	145.71	-150.59	0.68	58
**DHFR**	1M78	B	Ile112	CLZ	3.25	132.77	146.79	-156.55	0.68	58
**JNK3**	1PMN		Ala91	984	2.83	158.54	95.69	74.07	0.49	64
**GRP94**	1QYE		Gly153	CDY	2.92	141.50	101.13	-112.37	0.38	66
**Caspase-3**	1RHR		Gly287	CNE	3.11	143.61	106.47	-118.11	0.46	69
**RT**	1S1W		Leu234	UC1	3.23	156.48	93.94	81.11	0.85	70
**ENR**	2PD4	A	Ala95	DCN	3.04	167.54	121.35	175.60	0.05	71
**ENR**	2PD4	B	Ala95	DCN	3.05	167.73	122.73	172.01	0.09	71
**ENR**	2PD4	C	Ala95	DCN	3.05	168.34	122.62	169.52	0.09	71

Table S1. Relevant parameters of C=O/Cl halogen bonds formed in various protein/ligand systems (Continued)

Proteins	PDB ID	Chain	Res	Ligand	d	Θ1	Θ2	Ψ	strength	reference
**ENR**	2PD4	D	Ala95	DCN	3.02	166.33	120.72	179.61	0.03	71
**JNK3**	4Z9L		Ala91	880	2.94	149.99	95.94	80.21	0.62	64

ENR: Enoyl reductase; 3HNR: Trihydroxynaphthalene reductase; hEST: human Estrogen sulfotransferase; AR: Aldose reductase; PfDHFR-TS: Plasmodium falciparum dihydrofolate reductase–thymidylate synthase; hLGP: Human liver glycogen phosphorylase; F16BPase: Fructose-1,6-bisphosphatase; DHBD: 2,3-dihydroxybiphenyl 1,2-dioxygenase; DHFR: dihydrofolate reductase; JNK3: c-Jun N-terminal kinase 3; GRP94: endoplasmic reticulum paralog of cytoplasmic Hsp90; RT: HIV-1 reverse transcriptase

Table S2. Relevant parameters of C=O/Br halogen bonds formed in various protein/ligand systems.

Proteins	PDB ID	Chain	Res	Ligand	D	Θ1	Θ2	Ψ	strength	reference
**ART2.2**	1GXZ	A	Gln187	BRT	3.15	168.46	122.3	-128.98	0.57	77
**CK2**	1J91	B	Arg47	TBS	3.30	163.79	105.53	-103.52	0.85	75
**TTR**	1KGJ	C	Ala508	FL8	3.30	136.90	87.79	76.23	0.62	74
**TTR**	1KGJ	D	Ser717	FL8	3.35	150.10	102.18	77.08	0.85	74
**TK**	1KI4	A	His164	BTD	2.93	136.32	142.31	-56.58	0.67	72
**TK**	1KI4	B	His164	BTD	3.17	137.95	122.05	-72.7	0.86	72
**GPDH**	1N1G		Arg336	BCP	3.29	127.89	126.07	-146.47	0.43	76
**CDK2**	1P5E	A	Leu83	TBS	2.90	164.70	131.98	137.32	0.59	55
**CDK2**	1P5E	A	Ile10	TBS	3.24	144.08	110.05	-85.12	0.96	55
**CDK2**	1P5E	A	Glu81	TBS	3.01	168.55	154.87	-41.29	0.67	55
**CDK2**	1P5E	C	Glu81	TBS	3.05	171.72	151.49	-39.49	0.68	55
**CDK2**	1P5E	C	Leu83	TBS	2.88	176.94	121.18	127.42	0.47	55
**MDM2**	1RV1	A	Tyr100	IMZ	3.06	163.44	123.30	-137.19	0.46	79
**MDM2**	1RV1	B	Tyr100	IMZ	3.15	159.72	122.54	-136.73	0.46	79
**PPO**	1SEZ	B	Leu369	OMN	3.31	176.84	87.77	-72.99	0.58	78
**SD**	6STD	A	Gly165	MS2	3.26	165.01	86.29	-86.07	0.72	73
**SD**	6STD	B	Gly165	MS2	3.37	164.59	83.14	-86.07	0.69	73
**SD**	6STD	C	Gly165	MS2	3.26	162.67	86.92	-83.74	0.71	73

ART2.2: Ecto-ADP-ribosyl transferase; CK2: Casein Kinase 2; TTR: transthyretin; TK: Thymidine kinase; GPDH: glycerol-3-phosphate dehydrogenase; CDK2: cyclin-dependent protein kinase 2; MDM2: Ubiquitin-protein ligase E3 MDM2; PPO: Protoporphyrinogen IX oxidase; SD: scytalone dehydratase

Table S3. Relevant parameters of C=O/I halogen bonds formed in various protein/ligand systems.

Proteins	PDB ID	Chain	Res	Ligand	D	Θ1	Θ2	Ψ	strength	reference
**TTR**	1ETA	1	Ala109	T44	3.13	149.45	106.13	-89.20	0.93	57
**TTR**	1ETA	2	Ala109	T44	3.12	161.01	102.20	-84.21	0.88	57
**TTR**	1ETB	1	Thr109	T44	3.27	159.67	93.02	-87.27	0.75	57
**TTR**	1ETB	2	Thr109	T44	3.23	161.24	91.77	-88.94	0.70	57
**uPA**	1GJD		Val41	T44	3.42	163.88	156.85	-75.65	0.59	84
**HSA**	1HK2		Lys524	T44	3.22	143.89	113.73	-134.85	0.12	90
**HSA**	1HK2		Leu387	T44	3.34	162.08	129.90	-150.07	0.48	90
**HSA**	1HK2		Ile290	T44	3.29	152.67	129.56	-46.68	0.90	90
**HSA**	1HK3		Lys524	T44	3.14	143.18	119.84	-137.25	0.25	90
**HSA**	1HK3		Leu387	T44	3.48	169.30	127.41	-161.16	0.34	90
**HSA**	1HK3		Ile290	T44	3.41	153.71	133.50	-43.01	0.80	90
**HSA**	1HK4		Asn429	T44	3.21	132.53	113.58	-152.51	0.00	90
**HSA**	1HK5		Asn429	T44	3.47	132.52	111.11	-148.72	0.00	90
**TTR**	1ICT	D	Ser117	T44	3.46	131.88	91.39	69.93	0.50	86
**TTR**	1ICT	C	Ser117	T44	3.30	166.08	102.41	82.19	0.87	86
**TTR**	1ICT	A	Ala108	T44	3.24	149.27	72.25	69.75	0.00	86
**TTR**	1IE4		Ala109	T44	3.07	158.23	100.88	-82.25	0.82	87
**TTR**	1KGI	C	Ala509	T4A	3.07	130.34	95.47	-82.46	0.70	85
**TTR**	1KGI	D	Ala709	T4A	3.07	154.20	102.51	-97.51	0.72	85
**AK**	1LIJ		Thr45	RPP	3.34	149.10	107.75	-110.51	0.62	83

Table S3. Relevant parameters of C=O/I halogen bonds formed in various protein/ligand systems (Continued).

Proteins	PDB ID	Chain	Res	Ligand	D	Θ1	Θ2	Ψ	strength	Reference
**TRβ**	1NQ0		Phe272	4HY	3.33	173.15	124.28	-134.35	0.46	88
**TRβ**	1NQ2		Phe272	4HY	3.07	170.51	124.88	-134.21	0.44	89
**PHM**	1OPM		Ser190	IYG	3.40	152.24	97.59	78.99	0.78	81
**Cel6A**	1QK0		Ala180	IOB	3.41	148.50	134.83	-43.76	0.78	82
**PHM**	1SDW		Ser190	IYT	3.46	149.17	95.76	80.29	0.61	91
**TTR**	1THA	A	Ser117	T33	3.45	134.94	86.84	74.41	0.39	80
**TTR**	1THA	B	Ser117	T33	3.22	144.80	84.17	81.80	0.44	80

TTR: transthyretin; uPA: urokinase type plasminogen activator; HSA: human serum albumin; AK: adenosine kinase; TRβ: Thyroid hormone receptor β; PHM: Peptidylglycine α-hydroxylating monooxygenase; Cel6A: cellobiohydrolase Cel6A

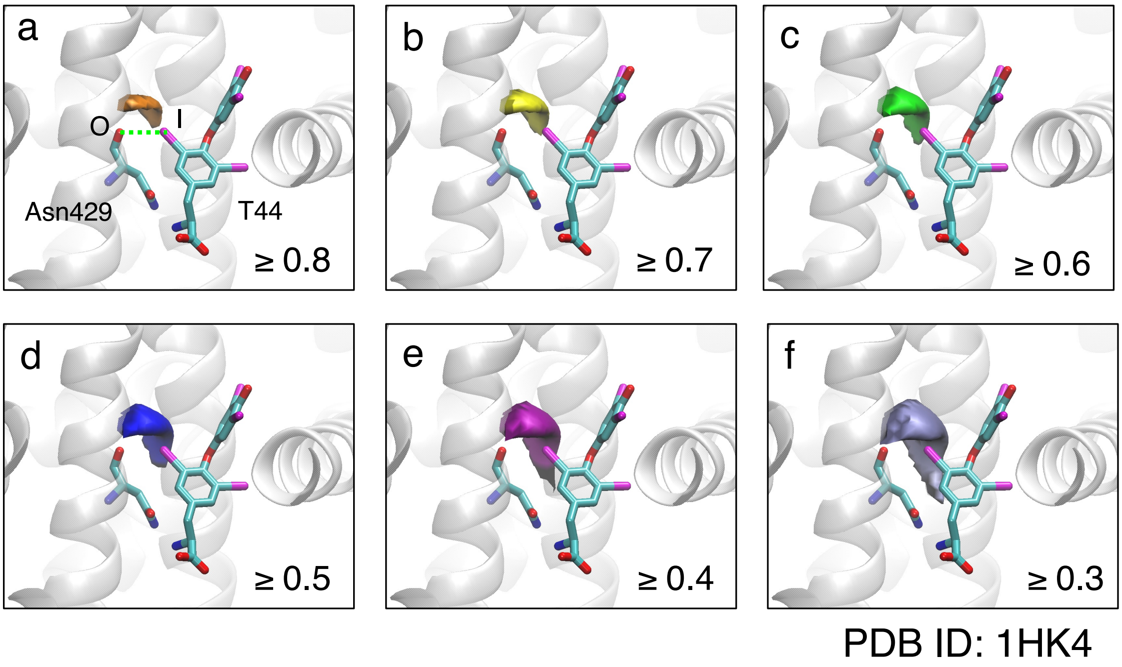

Figure S7. The ligand binding site of the human serum albumin (HSA)/ligand (T44) complex structure (PDB ID: 1HK4). The calculated MIF(func, I) are depicted by surface representations. (a) The halogen-bond-formable area with bond strengths of 0.8 or higher. The C-I/O halogen bond formed between the carbonyl oxygen of Asn429 and an iodine atom of T44 is represented by the dashed line. Halogen-bond-formable areas with bond strengths of (b) 0.7 or higher, (c) 0.6 or higher (d) 0.5 or higher (e) 0.4 or higher and (f) 0.3 or higher. For simplicity, only the effect of Asn429 was evaluated in the MIF(func, I) calculation.

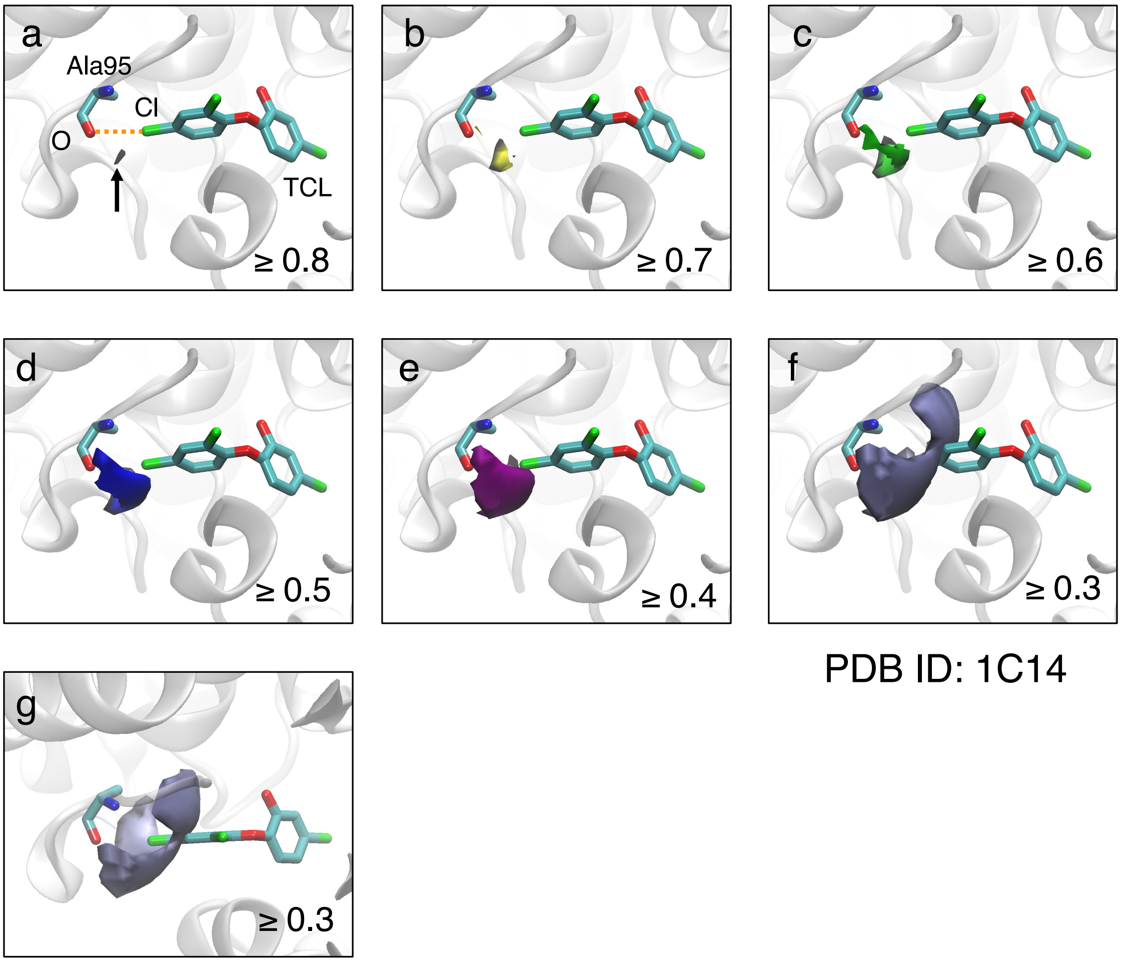

Figure S8. The ligand binding site of the enoyl reductase (ENR)/ligand (TCL) complex structure (PDB ID: 1C14). The calculated MIFs(func, Cl) are depicted by surface representations. (a) The halogen bond-formable area with bond strengths of 0.8 or higher. The C-Cl/O halogen bond formed between the carbonyl oxygen of Ala95 and a chloride atom of TCL is represented by the dashed line. Halogen-bond-formable areas with bond strengths of (b) 0.7 or higher, (c) 0.6 or higher (d) 0.5 or higher (e) 0.4 or higher and (f) 0.3 or higher. (g) Side view of (f). For simplicity, only the effect of Ala95 was evaluated in the MIF(func, Cl) calculation.

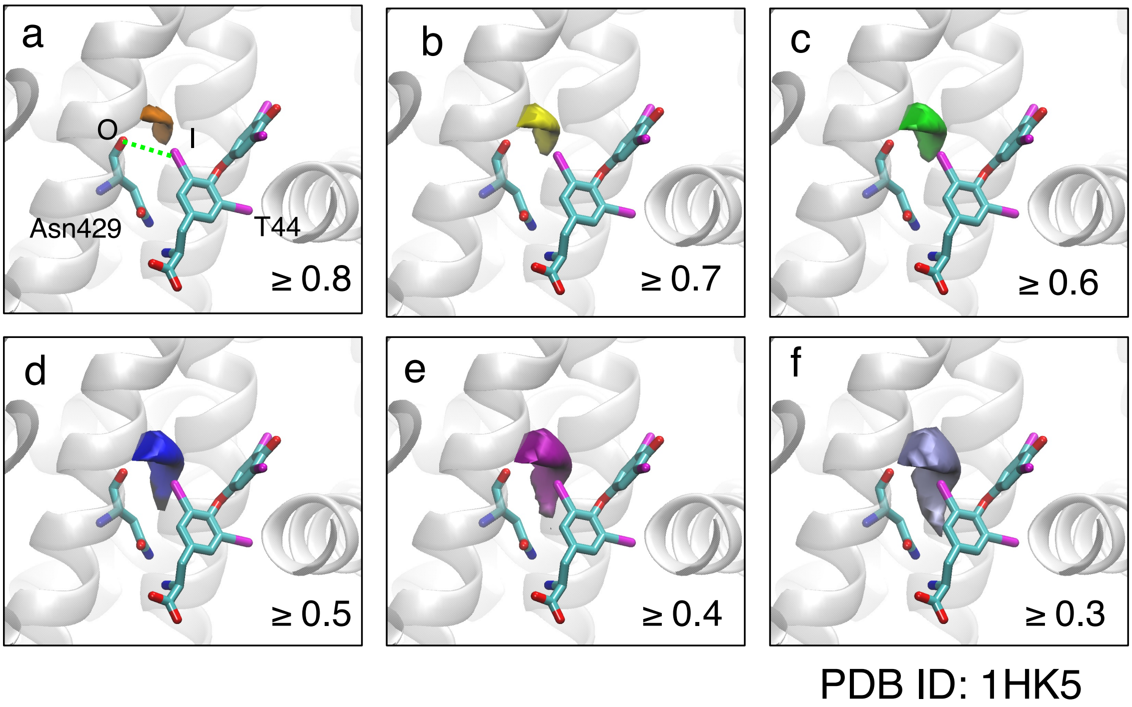

Figure S9. The ligand binding site of the human serum albumin (HSA)/ligand (T44) complex structure (PDB ID: 1HK5). The calculated MIF(func, I) are depicted by surface representations. (a) The halogen-bond-formable area with bond strengths of 0.8 or higher. The C-I/O halogen bond formed between the carbonyl oxygen of Asn429 and an iodine atom of T44 is represented by the dashed line. Halogen-bond-formable areas with bond strengths of (b) 0.7 or higher, (c) 0.6 or higher (d) 0.5 or higher (e) 0.4 or higher and (f) 0.3 or higher. For simplicity, only the effect of Asn429 was evaluated in the MIF(func, I) calculation.

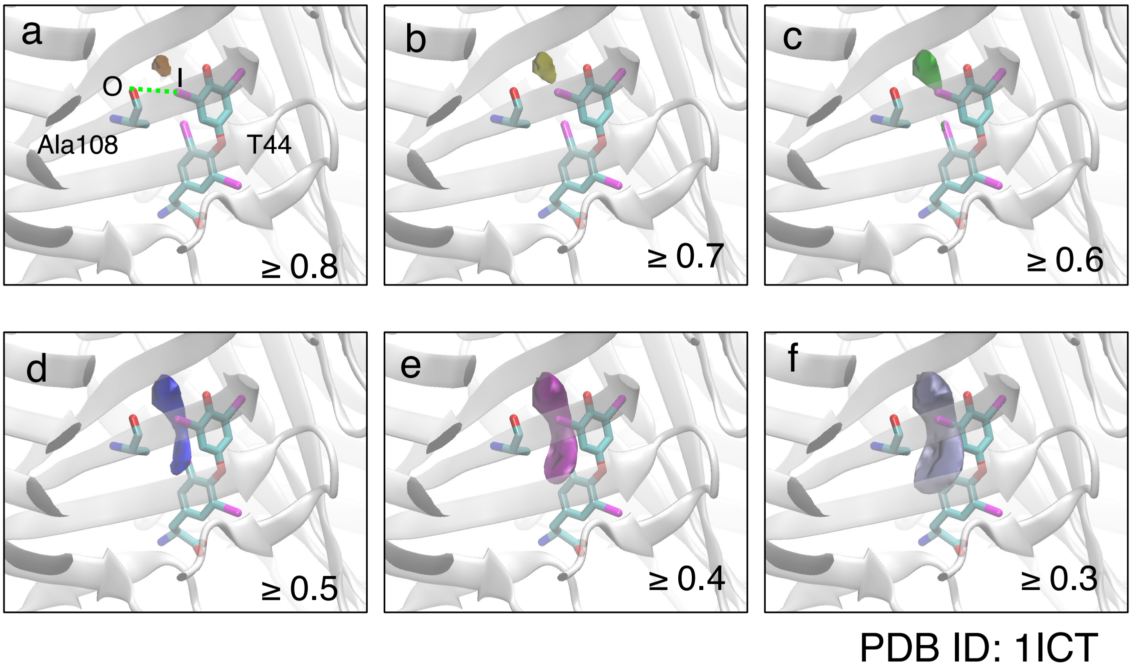

Figure S10. The ligand binding site of the transthyretin (TTR)/ligand (T44) complex structure (PDB ID: 1ICT). The calculated MIF(func., I) are shown by surface representations. The halogen bond formable area with a strength of 0.8 or higher is shown (a). The C-I/O halogen bond formed between the carbonyl oxygen of the Ala108 and an iodine atom of the T44 is shown by a dashed line. Likewise, the halogen bond formable area with a strength of 0.7 or higher (b), 0.6 or higher (c), 0.5 or higher (d), 0.4 or higher (e), and 0.3 or higher (f) are shown, respectively. For simplicity, only the effect of the Ala108 was evaluated in the MIF(func., I) calculation.
